# Bacteriological Monitoring of Inanimate Surfaces and Equipment in Some Referral Hospitals in Assiut City, Egypt

**DOI:** 10.1155/2019/5907507

**Published:** 2019-09-03

**Authors:** Entsar H. Ahmed, Hebat-Allah M. Hassan, Nahla M. El-Sherbiny, Asmaa M. A. Soliman

**Affiliations:** ^1^Medical Microbiology and Immunology Department, Assiut University, Asyut, Egypt; ^2^Public Health and Community Medicine Department, Faculty of Medicine, Assiut University, Asyut, Egypt

## Abstract

Hospital-acquired infections represent a serious public health problem in all countries. It is clear that monitoring of the hospital environment is an essential element in the control and a part of the policy for preventing nosocomial infections. It allows a better understanding of the microbial ecology for the purpose of conducting preventive and corrective actions. The aims of this work were to determine the percentage of bacterial contamination of environmental samples and to identify potential nosocomial pathogens isolated from environments of seven referral hospitals from 2009 to 2015. By using the swab technique, 12863 samples were collected. Qualitative and quantitative cultures were performed. The organisms were primarily identified by colony morphology, microscopy of Gram stain, and standard biochemical tests. 25.6% of total samples showed contamination (93% was monomicrobial and 7.0% was polymicrobial). The predominant species was coagulase-negative staphylococcus (CNS) (32%), followed by methicillin-resistant *S. aureus* (MRSA) (26%) and then *K*. *pneumonia* (10.6%). The percentage of contamination varied among the covered hospitals and according to the year of monitoring with highly statistically significant difference (*p* value < 0.001). Direct contact with environmental surfaces or equipment transmits the majority of nosocomial infection. Major nosocomial pathogens have been identified. Hospital managers and healthcare bodies must be aware of the reality of the concept of environmental bacterial tanks and the need for respect of biocleaning procedures and choice of biocleaning tools.

## 1. Introduction

Hospital or hospital-acquired infections represent a serious public health problem in all countries [1]. The burden of HAI is already substantial in developed countries, where it affects from 5% to 15% of hospitalized patients in regular wards and as many as 50% or more of patients in intensive care units (ICUs) [2, 3].

It is clear that monitoring of the hospital environment is an essential element in the control of nosocomial infections. As possible causes of infection, contamination of surfaces may be mentioned, even if cross contamination by hands is probably the greatest risk. In fact, hospital surfaces colonized by different types of microorganisms constitute special ecological niches that require cumbersome, complex, and costly procedures that are necessary for better safety of the patient [4].

There is a high prevalence of contamination of equipment and high-touch surfaces surrounding the patient [5]. The ability of microorganisms to survive on surfaces is due to their production of adhesion molecules and biofilms [6]. Direct contact primarily with environmental surfaces or equipment transmits the majority of nosocomial infection [7]. Major nosocomial pathogens have been identified. They can circulate between the patients and might persist in the environment for a long time [8].

As established by microbiological studies, certain hospital pathogens can survive on dry hospital surfaces for extended periods [9]. Both Gram-negative and Gram-positive bacteria can survive up to months on dry inanimate surfaces with longer persistence under lower temperature and humid condition [10].

Surfaces of commonly used medical equipment and high-contract communal surfaces (e.g., medical chart and telephones) could be contaminated by multidrug-resistant bacteria (MDR) [11]. In the issue of intensive care unit (ICU) where critically ill patients have several risk factors for nosocomial infection, the issue of environmental contamination poses an even greater challenge [12].

Monitoring of the hospital environment is lacking in hospitals in Assiut city, so the main objectives of this study were to determine the percentage of bacterial contamination of environmental samples and to identify potential nosocomial pathogens isolated from environments of seven referral hospitals in Assiut city as identification of bacterial contamination of environmental samples is a guide for appropriate preventive measures of infection.

## 2. Materials and Methods

This descriptive cross-sectional study was conducted in seven referral hospitals in Assiut city, Egypt, from 2009 to 2015. All hospitals belong to Assiut University Hospitals, except health insurance hospitals, as they are present in Assiut city, they are the most important hospitals in Assiut and for the whole upper Egypt, easy access, large drainage area for Assiut Governorate as well as for nearby Governorates and preferred to other hospitals due to highly qualified medical staff, good equipment, and facilities allowing good percentage of referral from near as well as distant areas. The selection of sampling sites was made in consultation with the heads of departments and targeted to the most representative and most critical location in each hospital. Random, undirected sampling was collected. Sterile swabs were moistened in sterile normal saline and rolled over the targeted inanimate surfaces/equipment separately (e.g., beds, ventilators, monitors, bedside tables, operation tables, anaesthesia equipment, trolley, dressings, bench, and surgical blades) [13]. Samples were transported to the Infection Control Laboratory of Assiut University Hospital. The swabs were cultured on blood agar plates at 35°C for 48 hours and subcultured on MacConkey' agar for the selection of Gram-negative bacteria [8].

Colonies were primarily identified by colony morphology, microscopy of Gram stain, and standard biochemical tests. [14] Only pathogenic microbes are examined.

### 2.1. Statistical Analysis

Data entry and analysis were performed using Statistical Package for Social Science version 14 (SPSS). *p* value was considered statistically significant when *p* < 0.05.

## 3. Results

**Figure 1:**
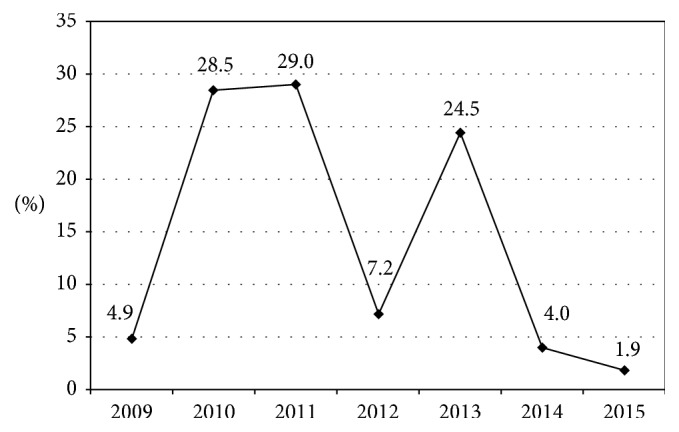
Distribution of the studied samples (2009–2015) in Assiut city.

**Figure 2:**
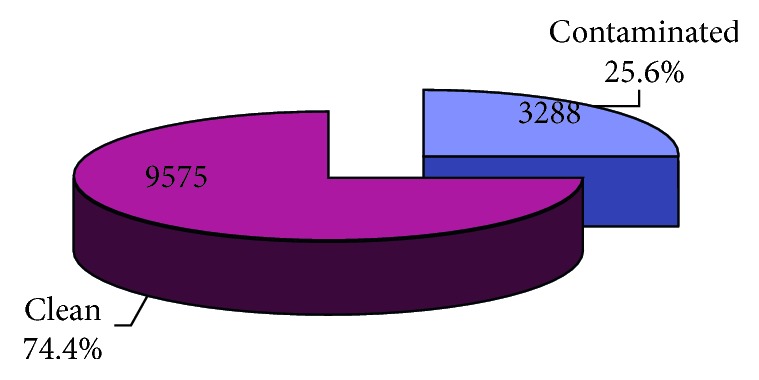
Percentage of contamination of the studied samples from different hospitals (2009–2015) in Assiut city.

**Figure 3:**
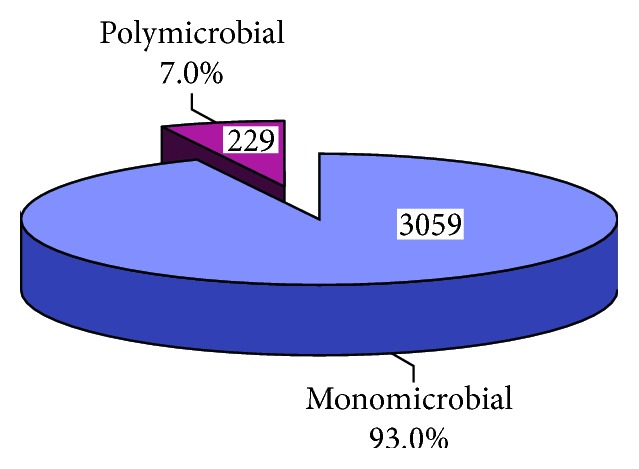
Pattern of contamination among different hospitals (2009–2015) in Assiut city.

A total of 12863 swab samples (covering different surface points/equipment) were collected in seven referral hospitals in Assiut city, Egypt, from 2009 to 2015. Most of the samples were collected in the years 2010 and 2011 and the least percentage (1.9%) in the year 2015 (Table 1 and Figure 1). More than half of the samples (57.5%) were collected from Main Assiut University Hospital and the least percentage (0.2%) from Urology University Hospital (Table 2). Contamination was positive in 25.6% of samples (Figure 2). According to the pattern of contamination, 7% of samples were polymicrobial (Figure 3). About 32% and 26% of isolated organisms were CNS and MRSA, respectively, followed by *K*. *pneumoniae* (20.7%) then Gram-positive bacillus sp. (*C. difficile*) (10.6%) (Table 3). In the year 2011, 31.0% of the samples were contaminated followed by the years 2015 and 2014 (26.9% and 26.7%), respectively, and the least contamination was in the year 2012 (9.0%) with statistically significant difference (*p* < 0.001) (Table 4).

At Main Assiut University Hospital, 30.5% of the samples were contaminated followed by Al-Ragehy University Hospital for liver and Neuropsychiatric University Hospital (26.6% and 25.3%), respectively, and the least contamination was at Pediatric University Hospital (15.5%) with statistically significant difference (*p* < 0.001) (Table 5).

**Figure 4:**
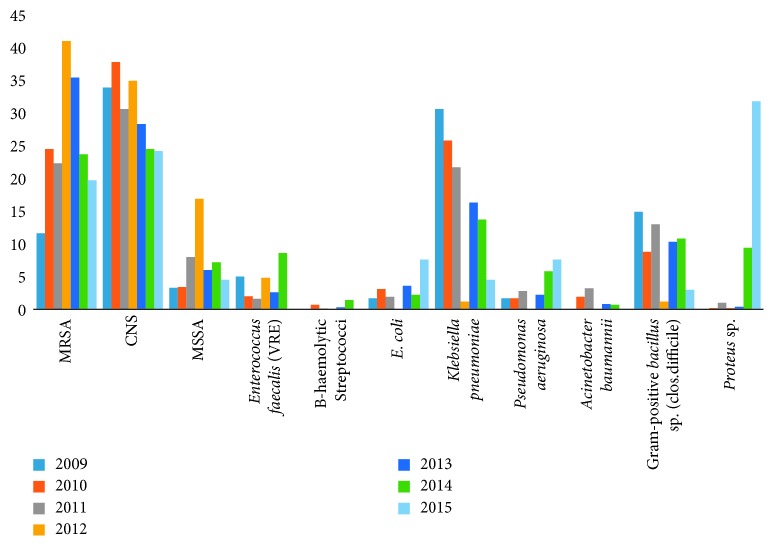
Distribution of the type of organism isolated according to the year of monitoring from different hospitals (2009–2015) in Assiut city.

For most of the years (2009–2012, 2014), the most common organism isolated was CNS (33.9%, 37.8%, 30.6%, 34.9%, and 24.5%), respectively (Figure 4). In Health Insurance Hospital, Pediatric University Hospital, Women Healthcare University Hospital, and Main Assiut University Hospital, the most common organism isolated was CNS (41.1%, 32.7%, 32.6%, 31.5%, and 24.5%), respectively (Table 6). The majority of organisms from all hospitals were monomicrobial (Table 7).

## 4. Discussion

In this study, we investigated a total of 12863 samples with 25.6% positivity of bacterial contamination. This was more or less similar to a study by Rozonska et al., as overall, 69.6% of samples exhibited growth of 19 bacterial species. Pathogenic species—representing indicator organisms of efficiency of hospital cleaning—was demonstrated by 21.4% of samples; among them, *Acinetobacter* spp., *Enterococcus* spp., and *Staphylococcus aureus* were identified. Coagulase-negative staphylococci (CNS) were predominant [15].

In another study, 51% of the environmental samples were contaminated with different bacterial species in the studied ICUs [16]. This was more or less twice the results of our study. Discrepancies between studies concerning the impact and degree of environmental contamination may reflect a complex epidemiology, differences in the measurement between studies, or the variable quality of institutional cleaning, which is an important and frequently unmeasured confounder [17]. Regarding the frequency of environmental contamination among hospitals under study, we noticed a wide range of variation that could be explained by the concept of Otter et al., who reported many factors such as method of sampling, organism culturability, and ease of particular environmental contamination (difficulty or cleaning) [9].

Multibacterial contamination of the environmental samples was estimated to be 11% [15]. This agrees with this study (7% polymicrobial). 10.6% of isolated organisms were *Clostridium difficile*. Spores of *Clostridium difficile* are durable and resistant to usual cleaning methods. Contamination of the inanimate environment by *C*. *difficile* has been reported to occur in areas in close proximity to infected or colonized patients [17].

Gram-negative bacilli: enteric Gram-negative bacilli are not commonly spread to patients from the dry inanimate environment; they are generally not viable after drying, lasting 7 h or less after desiccation [18].

Infection with these organisms is thought to occur because of endogenous spread or cross infection between patients via the hands of healthcare workers. However, *Pseudomonas aeruginosa* and *Acinetobacter baumannii* are strongly associated with environmental contamination.

Many studies have documented the contamination of sinks and sink drains by *P*. *aeruginosa* [17]. Rates of environmental contamination also vary on the basis of the site of infection in source patients: contamination is more common in the rooms of patients with urinary infection or wounds than in the rooms of patients with bacteremia only [19]. This study found that the proportion of various groups of bacteria significantly varied in respective hospitals.

Poor hygiene compliance facilitates more cross transmission and environmental contamination [20]. Absence of proper infection guidelines contributes to contamination of electrical equipment [21].

Hu et al. found difficulty in the eradication of environmental organisms and explained the survival of those organisms in dry surface biofilms [22].

In our study, CNS was found as the most common environmental isolate (31.9%) followed by MRSA, which was more or less agreed with Mohapatra et al., who reported that CoNs and MRSA are among the common environmental pathogens [23].

Gonsu et al. detected 148 bacterial isolates in inanimate surfaces in two referral hospitals, with CNS (57.43%) being the most predominant species [24].

These results are similar to those reported by Tagnouokam with a predominance of CNS (55%) [25]. This finding is also supported by another study as Lamali et al. found CNS (24%) positivity among environmental samples among a total of 81 investigated samples [4].

Also, these results were in agreement with Sauuide et al., where they found 26% positivity of CNS followed by MRSA 20% then *K. pneumonia* 16% [26]. In a study by Cordeiro et al., different species belonging to CNS were found in equipment before and after disinfection [27].

In the current study, *A. baumannii* was isolated from the hospital environments. This finding is supported by a similar study reported that *Acinetobacter* spp. remains in the hospital environment for a long time, transmitting the infection through staff hands and contaminated surfaces. [28].

We concluded that contamination of high-touch surfaces and equipment of hospital environments by pathogenic bacteria will raise the need for improving its biological safety and hand hygiene compliance of healthcare workers. These bacteria increase the risk of transmission to patient and subsequently hospital-acquired infection. We recommend periodic cultures to reduce the rate of contamination. Local epidemiology strategy is appropriate to address surface contamination.

## Figures and Tables

**Table 1:** Distribution of the studied samples (2009–2015) in Assiut city.

Year	Number (*n* = 12863)	%
2009	624	4.9
2010	3668	28.5
2011	3732	29.0
2012	922	7.2
2013	3152	24.5
2014	520	4.0
2015	245	1.9

**Table 2:** Distribution of the studied samples according to different hospitals (2009–2015) in Assiut city.

Hospital	Number (*n* = 12863)	%
Health Insurance Hospital	1064	8.3
Main Assiut University (MAU) Hospital	7397	57.5
Neuropsychiatric University Hospital	217	1.7
Pediatric University Hospital	2284	17.8
Al-Ragehy University Hospital for liver	252	2.0
Urology University Hospital	27	0.2
Women Healthcare University Hospital	1622	12.6

**Table 3:** Type of isolated organisms from different hospitals (2009–2015) in Assiut city.

	Number (*n* = 3288)	%
CNS (coagulase-negative staphylococci)	1051	31.9
MRSA	858	26
*Klebsiella pneumoniae*	682	20.7
Gram-positive bacillus sp. (*C. difficile*)	350	10.6
MSSA (methicillin-sensitive *S. aureus*).	202	6.1
*E. coli*	89	2.7
VRE (vancomycin-resistant enterococci)	80	2.4
*Pseudomonas aeruginosa*	80	2.4
*Acinetobacter baumannii*	62	1.8
*Proteus* sp.	51	1.5
B-Haemolytic Streptococci	12	0.36
*Candida albicans*	7	0.21

**Table 4:** Distribution of contamination according to the year of monitoring from different hospitals (2009–2015) in Assiut city.

	Year													
Contamination	2009		2010		2011		2012		2013		2014		2015	
	Number	%	Number	%	Number	%	Number	%	Number	%	Number	%	Number	%
Contaminated	121	19.4	948	25.8	1158	31.0	83	9.0	773	24.5	139	26.7	66	26.9
Clean	503	80.6	2720	74.2	2574	69.0	839	91.0	2379	75.5	381	73.3	179	73.1
*p* value	<0.001^*∗*^													

**Table 5:** Distribution of contamination among different hospitals (2009–2015) in Assiut city.

	Hospital													
Contamination	Health Insurance Hospital		Main Assiut University Hospital		Neuropsychiatric University Hospital		Pediatric University Hospital		Al-Ragehy University Hospital		Urology University Hospital		Women Healthcare University Hospital	
	No.	%	No.	%	No.	%	No.	%	No.	%	No.	%	No.	%
Contaminated	219	20.6	2259	30.5	55	25.3	355	15.5	67	26.6	5	18.5	328	20.2
Clean	845	79.4	5138	69.5	162	74.7	1929	84.5	185	73.4	22	81.5	1294	79.8
*p* value	<0.001^*∗*^													

**Table 6:** Distribution of the type of organism isolated according to monitoring from different hospitals (2009–2015) in Assiut city.

Organism	Hospital													
	Health Insurance Hospital		Main Assiut University Hospital		Neuropsychiatric University Hospital		Pediatric University Hospital		Al-Ragehy University Hospital		Urology University Hospital		Women Healthcare University Hospital	
	No.	%	No.	%	No.	%	No.	%	No.	%	No.	%	No.	%
MRSA	57	26.0	609	27.0	6	10.9	65	18.3	26	38.8	3	60.0	92	28.0
CNS	90	41.1	711	31.5	9	16.4	116	32.7	18	26.9	0	0.0	107	32.6
MSSA	19	8.7	109	4.8	0	0.0	25	7.0	5	7.5	0	0.0	44	13.4
*Enterococcus faecalis* (VRE)	6	2.7	49	2.2	0	0.0	11	3.1	5	7.5	0	0.0	9	2.7
B-Haemolytic streptococci	5	2.3	5	0.2	0	0.0	0	0.0	2	3.0	0	0.0	0	0.0
*E*. *coli*	1	0.5	61	2.7	6	10.9	11	3.1	2	3.0	0	0.0	8	2.4
*Klebsiella pneumoniae*	18	8.2	522	23.1	3	5.5	92	25.9	0	0.0	1	20.0	46	14.0
*Pseudomonas aeruginosa*	5	2.3	45	2.0	4	7.3	7	2.0	6	9.0	0	0.0	13	4.0
*Acinetobacter baumannii*	0	0.0	60	2.7	0	0.0	2	0.6	0	0.0	0	0.0	0	0.0
Gram-positive bacillus sp. (*C*. *difficile*)	8	3.7	275	12.2	12	21.8	35	9.9	5	7.5	1	20.0	14	4.3
*Proteus* sp.	16	7.3	16	0.7	15	27.3	3	0.8	1	1.5	0	0.0	0	0.0

**Table 7:** Pattern of contamination among different hospitals.

Number of organisms	Hospital													
	Health Insurance Hospital		Main AUH		Neuropsychiatric University Hospital		Pediatric University Hospital		Al-Ragehy University Hospital		Urology University Hospital		Women Healthcare University Hospital	
	No.	%	No.	%	No.	%	No.	%	No.	%	No.	%	No.	%
Monomicrobial	212	96.8	2059	91.1	55	100.0	343	96.6	64	95.5	5	100.0	321	97.9
Polymicrobial	7	3.2	200	8.9	0	0.0	12	3.4	3	4.5	0	0.0	7	2.1

## Data Availability

The data used to support the findings of this study are available from the corresponding author upon request.
